# Circulating ciRS-7 as a potential non-invasive biomarker for epithelial ovarian cancer: An investigative study

**DOI:** 10.1016/j.ncrna.2022.07.004

**Published:** 2022-07-31

**Authors:** Aferin Beilerli, Sema Begliarzade, Albert Sufianov, Tatiana Ilyasova, Yanchao Liang, Ozal Beylerli

**Affiliations:** a Department of Obstetrics and Gynecology, The 2nd Affiliated Hospital of Harbin Medical University, Harbin, Heilongjiang, China; b Republican Clinical Perinatal Center, Ufa, Republic of Bashkortostan, 450106, Russia; c Department of Neurosurgery, Sechenov First Moscow State Medical University (Sechenov University), Moscow, Russia; d Department of Internal Diseases, Bashkir State Medical University, Ufa, Republic of Bashkortostan, 450008, Russia; e Department of Neurosurgery, the First Affiliated Hospital of Harbin Medical University, Harbin, 150001, China; f Educational and Scientific Institute of Neurosurgery, Рeoples’ Friendship University of Russia (RUDN University), Moscow, Russia

**Keywords:** Ovarian cancer, Epithelial ovarian cancer, ciRS-7, Hsa-miR-7-5p, Circulating, Diagnosis, Prognosis

## Abstract

**Background:**

Ovarian cancer (OC) is the most common malignant neoplasm of the female reproductive system in developed countries. Early detection, diagnosis and prognosis are particularly important to OC. The potential of circulating circular RNAs (circRNAs) as non-invasive biomarkers of various tumors has been especially described in recent years. The aim of this study was to explore the diagnostic and prognostic value of circulating cirRS-7 in patients with epithelial ovarian cancer (EOC).

**Methods:**

Pre- and postoperative plasma samples from 111 EOC patients (47 cases with FIGO stage IA-IIB and 64 cases with FIGO stage IIB-IV) and healthy female volunteers was collected. Circulating ciRS-7 and hsa-miR-7-5p was analyzed using reverse transcription polymerase chain reaction (qRT-PCR). The diagnostic and prognostic value of circulating cirRS-7 as biomarker was estimated by the Receiver Operating Characteristic (ROC) curve and the area under the curve (AUC) and Kaplan–Meier analysis.

**Results:**

The preoperative expression levels of circulating ciRS-7 were increased in plasma of EOC FIGO stage I-IV patients than in the healthy controls (p < 0.001). However, the expression levels of ciRS-7 in the postoperative period were significantly lower in EOC FIGO stage IIA-IIA patients than healthy controls and EOC FIGO stage IIB-IV patients (p < 0.05, p < 0.001). The AUC of ciRS-7 for diagnosing EOC FIGO stage I-IV patients in pre-and postoperative periods was 0.90, 0.92, 0.84, 0.88, 0.58 and 0.86, respectively. Higher circulating ciRS-7 expression is associated with lymph node invasion, FIGO stage, distant metastasis, and worse overall survival (OS) of patients. Moreover, multivariate Cox analysis showed that higher circulating ciRS-7 was an independent predictor of OS in EOC FIGO stage IIB-IV patients. In addition, in plasma of EOC patients, ciRS-7 negatively correlated with has-miR-7-5p in pre-and postoperative periods (p < 0.001).

**Conclusions:**

Circulating ciRS-7 levels in plasma can be considered a potential candidate biomarker for diagnosing EOC patients. Dysregulation of ciRS-7 may participate in the molecular mechanism of EOC through hsa-miR-7-5p sponging.

## 1 Introduction

Ovarian cancer (OC) is one of the most common cancers in women reproductive system. Most OCs are classified as epithelial ovarian cancer (EOC). In fact, EOC account for 80%–90% of all OCs. Approximately 220,000 new cases of OC have been registered in the world annually [1]. This tumor is characterized by high mortality during the first year after diagnosis (∼25%) and the highest mortality among all gynecological malignant tumors [1,2]. The five-year survival rate for OC is less than 40%. However, more than 80% of patients with advanced OC will relapse, and with the associated with a very poor prognosis [3]. For this tumor, the asymptomatic course is specific, and therefore the diagnosis is made mainly at IIB-IV stages (FIGO staging system), after the extensive spread of the tumor in the abdominal cavity, which limits the effectiveness of surgery and chemotherapy [3]. Currently, modern treatment of OC is based on cytoreductive tumor surgery, in which, with the help of several courses of combined chemotherapy (e.g., paclitaxel + cisplatin), the rate of complete remission can reach 80% [4]. However, the high relapse rate and high drug resistance after relapse are among the most important causes of high mortality from this disease. The main cause of death from OC is treatment-resistant metastases [3]. Therefore, early diagnosis, prognosis and new methods of treatment for OC are an urgent problem that needs to be addressed. The existence of circRNAs in human body fluids (e.g. whole blood and plasma/serum) was identified; this brings a new field for the potential function for circRNAs as non-invasive biomarkers in human disease, especially in human malignant tumors [[4], [5], [6], [7]].

Numerous studies have also demonstrated that ciRS-7 (also known as CDR1as) play an important regulatory role in the development and progression of various types of human tumors, particularly in non-small cell lung cancer (NSCLC), gastric cancer (GC), clear cell renal cell carcinoma (ccRCC) and so on [[8], [9], [10]]. For example, one of the studies has confirmed that ciR-7 can interact with miR-7 and nuclear factor-kappa B (NF-κB) involved in NSCLC, thus, participating in NSCLC cell proliferation, invasion, apoptosis, and migration [8]. Zhang et al. demonstrated that ciRS-7 acted as a sponge of miR135a-5p, and reversed miR-135a-5p-mediated downregulation of oncogene transient receptor potential cation channel subfamily C member 1 (TRPC1). Importantly, the authors found that the expression levels of ciRS-7 in GC samples was associated with overall survival (OS) and disease-free survival (DFS) [9]. In other study, ciRS-7 up-regulation in human ccRCC tissues could promote ccRCC cell proliferation and invasion via a regulated epidermal growth factor receptor (EGFR)/Akt signaling pathway [10]. In addition, the same features observed in these tumors are all present in OC. For instance, Zhang et al. found that the ciRS-7 promoted tumor cell growth and metastasis in OC, via regulating zinc finger E-box binding homeobox 1 (ZEB1) and mouse double minute 2 homolog (MDM2)-mediated epithelial–mesenchymal transition (EMT) by sponging miR-641. Thus, ciRS-7 may play an important role in OC development [11]. However, to our knowledge, the non-invasive biomarker‐based investigation focusing on circulating circRNAs including ciRS-7 in human OC was poorly investigated.

In this study, we revealed the landscape of circulating ciRS-7 through the reverse transcription polymerase chain reaction (qRT-PCR) technology in EOC patients before and after operation, and healthy controls. The diagnostic and prognostic value of circulating cirRS-7 as biomarker was estimated by the Receiver Operating Characteristic (ROC) curve and Kaplan–Meier analysis. We especially wanted to investigate the expression levels of circulating ciRS-7 with regard to FIGO stages and lymph node and distant metastasis status. In addition, we explored the link between ciRS-7 and hsa-miR-7 in plasma from EOC patients.

## 2 Materials and methods

### 2.1 Study population and sample collection

Blood samples utilized in this study were collected between 2018 and 2020 from 111 EOC patients at the Republican Clinical Oncological Dispensary. Pathological characterization of tumor stages was assessed according to the FIGO and LNM criteria. The patients we assayed were in different FIGO stages of EOC, of which 47 were in stage IA-IIA (early stage) and 64 in stage IIB-IV (advanced stage). The diagnosis of each case was confirmed through histological examination. None of the patients had a medical history of immune diseases, organ failure, injuries, infections, other tumors or metastatic tumors from other sites or had received chemo- or radiotherapy prior to plasma samples collection. Paired preoperative and postoperative blood samples (n = 111) were collected from the same patients before surgery and on the 7th day after resection. Blood samples were also collected from 77 healthy female volunteers (healthy controls). The study protocol was approved by the ethics committee of the Republican Clinical Oncological Dispensary and implemented in accordance with the principles of the Helsinki Declaration. Written informed consent was obtained from all participants. Clinical characteristics of the EOC patients are summarized in Table 1.

**Table 1:** The baseline information of epithelial ovarian cancer (EOC) patients (n = 111).

Characteristics	Numbers of cases, n (%)
Age (years)	
<50	42 (46.6)
≥50	69 (53.4)
Preoperative serum CA125 (ng/mL)	
<100	36 (16.7)
≥100	75 (83.3)
Tumor size (cm)	
<2	60 (66.6)
≥2	51 (33.4)
FIGO stage	
IA-IIA	47 (52.2)
IIB-IV	64 (47.8)
Ascites (mL)	
<1000	22 (24.4)
≥1000	42 (46.6)
LNM	
Negative	60 (66.6)
Positive	51 (33.4)
Distant metastasis	
Liver	29 (32.2)
Lung	14 (15.5)

**Abbreviations:** LNM, Lymph node metastasis; CA 125, Cancer antigen 125; EOC, Epithelial ovarian cancer.

### 2.2 Plasma preparation

All plasma samples were extracted from ethylenediaminetetraacetic acid (EDTA) tubes and centrifuged as described previously [12,13]. After the first centrifugation for 10 min at 1600 g, the supernatants were carefully removed and transferred to a new tube follow by centrifugation again at 16,000 g for 10 min to remove residual blood cells. Plasma was then stored at −80 °C until further processing.

### 2.3 Total RNA extraction

Total RNA was extracted from 200 μL plasma samples of OC patients and healthy controls using the miRNeasy Serum/Plasma Kit for purification of total RNA, including miRNA (Qiagen, Germany) and QIAzol Lysis Reagent (Qiagen, Germany) according to the manufacturer's instructions. The proposed method for the extraction of total RNA from plasma is as follows. A five times volume of QIAzol Lysis Reagent and an equal volume of chloroform were added to the plasma sample, then centrifuged for 15 min at 12,000 g at 4 °C. One and a half times the volume of 97–100% ethanol was added to the resulting double supernatant and applied to mini-columns from the miRNeasy Serum/Plasma Kit (QIAGEN), washing the membrane from unbound biopolymers and chemical agents. The sorbent was washed to remove impurities of non-nucleotide biopolymers and chemical agents RWT and RPE with buffer solutions according to the manufacturer's instructions. After that, the total RNA were eluted with 14 μm RNase-free water from the sorbent, with incubation for 10 min on ice between the elution steps. RNA purity and concentration were determined using a Nano-Drop 2000 (Thermo Scientific) and consistently yielded A260:A280 and A260:A230 ratios close to 2.0. All isolated total RNA was stored at a −80 °C freezer until use.

### 2.4 Complementary DNA (cDNA) synthesis and reverse transcription polymerase chain reaction (qRT-PCR)

Complementary DNA (cDNA) was synthesized using Transcriptor First Strand cDNA Synthesis Kit (Roche, Germany) by reverse transcription according to the manufacturer's instructions. qRT-PCR was carried out following the manufacturer's protocol of FastStart Universal SYBR Green Master (Rox) (Roche, Germany) with 2 μL cDNA template. The PCR mixture (18 μL) contains 10 μL SYBR Green (Rox) (Roche, Germany), 1 μL 10 mmol/mL forward primer (Invitrogen, USA), 1 μL 10 mmol/mL reverse primer (Invitrogen, USA) and 6 μL DEPC water (Invitrogen, USA). PCR reaction was performed in duplicates. All PCR reactions were carried out on an ABI 7500 Real-Time PCR machine (Thermo Fishers, USA). Reaction conditions were 95 °C for 10 min, followed by 40 cycles of 95 °C for 10 s and 60 °C for 10 s. Glyceraldehyde 3-phosphate dehydrogenase (GAPDH) and U6 snRNA was used as an endogenous control that is stably expressed across samples. The sequence of all primers used in this study is provided in Table 2.

**Table 2:** Sequence of all primers.

ncRNA/endogenous control gene	Primer Sequence (5′-3′)
ciRS-7	F: ACG TCT CCA GTG TGC TGA R: CTT GAC ACA GGT GCC ATC
hsa-miR-7-5p	F: CTA GCT AGC TAG AGC ACC AAT AGG GAA GGG R: GAA GAT CTT CGA GTC TGC CGA TGG GTG T
U6	F: CTC GCT TCG GCA GCA CA R: AAC GCT TCA CGA ATT TGC GT
GAPDH	F: GAC TCA TGA CCA CAG TCC ATG C R: AGA GGC AGG GAT GAT GTT CTG

**Abbreviations:** ncRNA, non-coding RNA; GAPDH, glyceraldehyde 3-phosphate dehydrogenase.

### 2.5 Statistical analysis

Relative levels of the circulating ciRS-7 and hsa-miR-7-5p were quantified using the 2-ΔΔCq method. ROC curves and the area under the curve (AUC) was applied to analysis the diagnostic values of the circulating ciRS-7. Kaplan–Meier analysis was used to generate and analyze survival time data. The univariate Cox proportional hazards regression was used for univariate and multivariate analyses. The Student t-test, ANOVA, chi-square analysis, or Mann-Whitney test was applied, where appropriate. A probability of p < 0.05 (*) or p < 0.001 (**) was considered statistically significant. The statistical analyses were carried out with the IBM SPSS 13.0 software and the graphs were generated by using Graphpad Prism 7.0.

## 3 Results

### 3.1 CiRS-7 expression in plasma from EOC patients and healthy controls

**Fig. 1:**
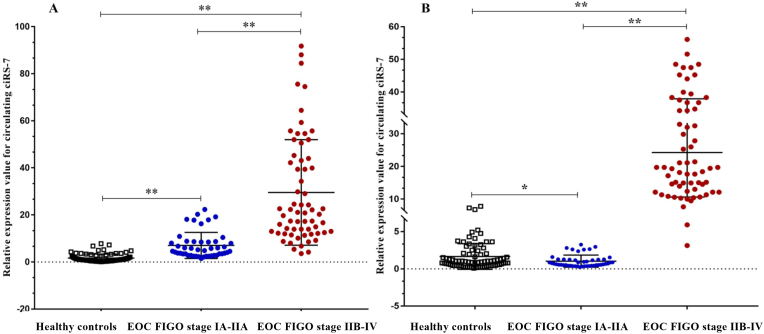
Quantification of the change in the expression levels of circulating ciRS-7 in the plasma of patients with EOC FIGO stage IA-IIA (n = 47) and patients with EOC FIGO stage IIB-IV (n = 64) in pre - and postoperative periods and healthy controls (n = 77). **(A)** Relative circulating ciRS-7 expression was statistically higher in EOC FIGO stage IA-IIA preoperative samples and **(B)** statistically lower in postoperative samples than in the healthy controls detected by reverse transcription polymerase chain reaction (qRT-PCR). **(A)** Relative circulating ciRS-7 expression in pre – and **(B)** postoperative EOC plasma samples from FIGO stage IIB-IV patients was statistically significantly higher than in the healthy controls and EOC FIGO stage IA-IIA patients detected by qRT-PCR.

In this study, we found that the expression preoperative levels of plasma ciRS-7 were higher in the EOC FIGO stage IA-IIA patients group and significantly higher in the EOC FIGO stage IIB-IV patients group than in the healthy controls (Fig. 1A; p < 0.001). Furthermore, we saw that in the postoperative period the expression levels of plasma ciRS-7 were significantly lower in the EOC FIGO stage IA-IIA patients group; in the EOC FIGO stage IIB-IV patients group there was only a slight decrease in the expression levels of circulating ciRS-7 than in the healthy controls (Fig. 1B; p < 0.05, p < 0.001). In addition, there was a statistically significant correlation between changes in the expression levels of plasma ciRS-7 between EOC FIGO stage IA-IIA and EOC FIGO stage IIB-IV patients in pre- and postoperative periods (Fig. 1A and B; p < 0.001). Thus, these results indicated that the circulating ciRS-7 expression was higher in plasma EOC patients, which may be involved in tumor progression, particularly in advanced-stage of EOC.

**Fig. 2:**
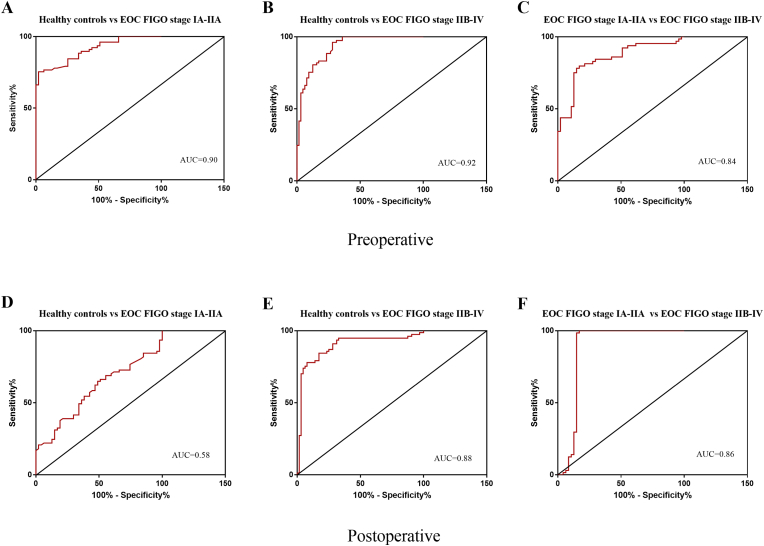
Receiver Operating Characteristic (ROC) curve analysis of circulating ciRS-7 in plasma from patients with EOC FIGO stage IA-IIA (n = 47) and patients with EOC FIGO stage IIB-IV (n = 64) in pre - and postoperative periods and healthy controls (n = 77). **(A, B)** Circulating ciRS-7 was able to differentiate EOC FIGO stage I–V patients from healthy controls in preoperative periods; **(E)** Circulating ciRS-7 was able to differentiate EOC FIGO stage IIB-IV patients from healthy controls in postoperative periods; **(C, F)** Circulating ciRS-7 was able to differentiate EOC FIGO stage IIB-IV patients from EOC FIGO stage IIA-IIA patients in pre-and postoperative periods.

The diagnostic value in pre- and postoperative periods of circulating ciRS-7 in differentiating the EOC FIGO stage I–V patients group from the healthy control group (AUC = 0.90 [95% confidence interval (CI): 0.855–0.955], AUC = 0.90 [95% CI: 0.879–0.966], AUC = 0.58 [95% CI: 0.478–0.678], and AUC = 0.88 [95% CI: 0.829–0,949]) and the EOC FIGO stage IA-IIA patients group from the EOC FIGO stages IIB-IV patients group (AUC = 0.84 [95% CI: 0.763–0.914] and AUC = 0.86 [95% CI: 0.769 to 0.958]) was also examined (Fig. 2A–F). These findings suggest that plasma ciRS-7 had high power to distinguish EOC FIGO stage I-IV patients group from healthy controls, especially in preoperative period (Fig. 2A and B). In addition, ROC curve analysis demonstrated that the circulating ciRS-7 displayed considerable accuracy in discriminating of EOC FIGO stage IA-IIA patients group from EOC FIGO stages IIB-IV patients group in the pre- and postoperative periods (Fig. 2C and F).

### 3.2 The high expression level of ciRS-7 associated with lymph node and distant metastasis status in EOC patients

**Fig. 3:**
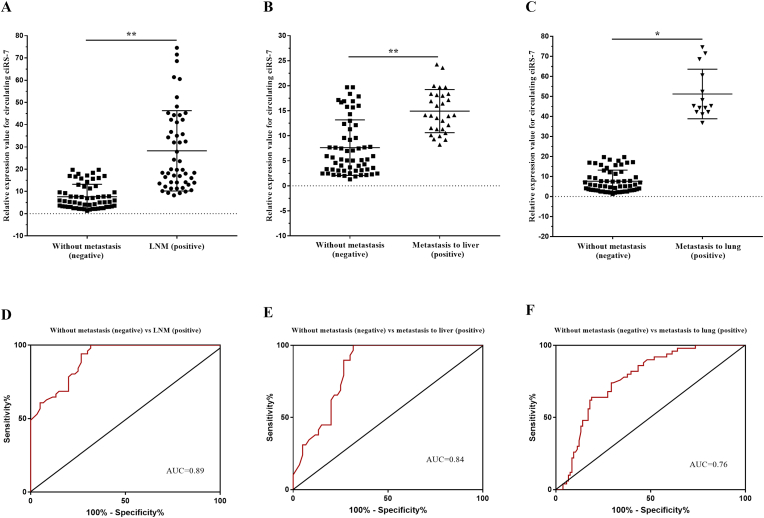
Circulating ciRS-7 as an independent prognostic factor for lymph node and distant metastasis status and Receiver Operating Characteristic (ROC) curve analysis based on the preoperative circulating ciRS-7-based signature in the lymph node metastasis cohort and distant metastasis cohorts. **(A)** Relative circulating ciRS-7 expression level was statistically significantly higher in EOC plasma samples of the positive lymph node metastasis group (n = 51), (B) in EOC plasma samples of the positive metastasis to liver group (n = 29) and (C) in EOC plasma samples of the positive metastasis to lung group (n = 14) than in the negative without metastasis group (n = 60). Circulating ciRS-7 was able to differentiate patients with **(D)** lymph node metastasis, **(E)** metastasis to liver and to lung **(F)** from EOC patients without metastasis (negative).

Furthermore, we analyzed the association between circulating ciRS-7 expression and lymph node and distant metastasis of EOC patients. We showed that the circulating ciRS-7 expression level in pre-and postoperative periods was significantly increased in the EOC FIGO stages IIB-IV patients group than in the EOC FIGO stages IIA-IIA patients group and healthy controls (Fig. 1A and B). To explore whether the high circulating ciRS-7 expression level is correlated with metastasis status in the EOC patients with advanced-stage, we next examined the expression patterns of circulating ciRS-7 in the plasma (preoperative) of 51 EOC patients with lymph node and distant metastasis status. Our results found that the circulating ciRS-7 expression level was significantly higher in the EOC patients with lymph node metastasis (n = 51) than that in the EOC without metastasis (n = 60) (Fig. 3A; p < 0.001), also significantly higher in the EOC patients with distant metastasis, particularly to liver (n = 29) (Fig. 3B; p < 0.001) and lung (n = 14) (Fig. 3C; p < 0.05), than that in the EOC without metastasis (n = 60) (Fig. 3A; p < 0.001). Our ROC curve analysis also revealed that plasma ciRS-7 levels significantly differentiate patients who had EOC with lymph node metastasis (AUC = 0.90 [95% CI: 0.859–0.959]), liver metastasis (AUC = 0.84 [95% CI: 0.762–0.921]), or lung metastasis (AUC = 0.76 [95% CI: 0.696–0.844]), respectively (Fig. 3D–F). Thus, these results suggesting that ciRS-7 plays a prospective oncogenic function in EOC and indicated that circulating cirRS-7 is a potential independent predictor factor for metastasis in patients with EOC.

### 3.3 Higher ciRS-7 expression predicts a poor prognosis of EOC patients

**Fig. 4:**
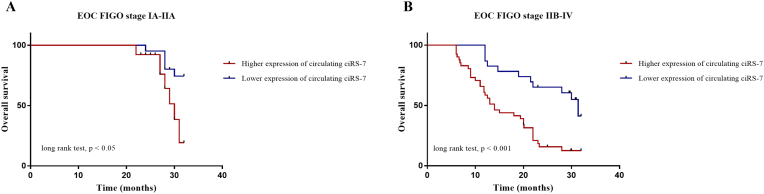
Kaplan–Meier curves of overall survival (OS) for epithelial ovarian cancer (EOC) (n = 111) patients based on the circulating ciRS-7 signature in FIGO stage IA-IIA (n = 47) **(A)** and FIGO stage IB-IV (n = 64) **(B).**

The prognostic value of circulating ciRS-7-based signature in OS was detectable through the Kaplan–Meier curve of two cohorts of EOC patients as shown in Fig. 4. The relative expression of circulating ciRS-7 in EOC FIGO stage IA-IIA patients and EOC FIGO stage IIB-IV patients were divided into a higher-expression group and a lower-expression group. Our analysis showed that EOC FIGO stage IIB-IV patients in the higher-expression group of circulating ciRS-7 had a poorer OS (Fig. 4B; p < 0.001) than in the EOC FIGO stage IA-IIA patients (Fig. 4A; p < 0.05). Next, to develop a circulating ciRS-7-based prognosis model, we used univariate and multivariate COX analysis to identify risk factors (Table 3). Based on these findings, we suggest that circulating ciRS-7 expression can be used as an independent factor to predict the survival of patients with advanced-stage of EOC.

**Table 3:** The univariate and multivariable Cox proportional hazards regression in EOC FIGO stage IIB-IV patients.

Variables	Univariate Cox analysis		Multivariate Cox analysis	
	HR (95% CI)	p-value	HR (95% CI)	p-value
Relative expression of ciRS-7	1.047 (1.031–1.063)	<0.001	1.021 (1.004–1.037)	0.02
Age (years)	2.675 (1.244–4.342)	<0.001	2.584 (1.884–3.996)	0.002
Tumor size (cm)	2.29 (0.69–7.59)	0.18	2.465 (1.611–3.588)	<0.001
LNM	2.89 (0.83, 10.05)	0.01	2.672 (1.198–5.958)	0.04
Distant metastasis	2.155 (1.499–3.356)	<0.001	3.987 (2.738–5.806)	<0.001
Ascites	1.893 (0.978–4.072)	0.07	1.18 (0.33, 4.25)	0.80
Serum CA125	0.813 (0.530–1.249)	0.35	0.907 (0.579–1.420)	0.67

**Abbreviations:** LNM, Lymph node metastasis; CA 125, Cancer antigen 125; EOC, Epithelial ovarian cancer; HR, hazard ratio; CI, confidence interval.

### 3.4 Interaction between ciRS-7 and miRNAs

Using miRNA target prediction software targetScan, miRanda and circular RNA Interactome, we investigated the top 3 predicted miRNAs targets for ciRS-7. The candidate ciRS-7 is predicted to harbour sites for hsa-miR-7-5, hsa-miR-6766–3p, and hsa-miR-490–5p, with different seed region types (i.e., 8mer, 7mer-m8, and 7mer-1A, respectively). We chose has-miR-7-5p for detailed investigation of a correlation with circulating ciRS-7 in plasma.

### 3.5 Correlation of ciRS-7 expression with hsa-miR-7-5p in plasma of EOC patients

**Fig. 5:**
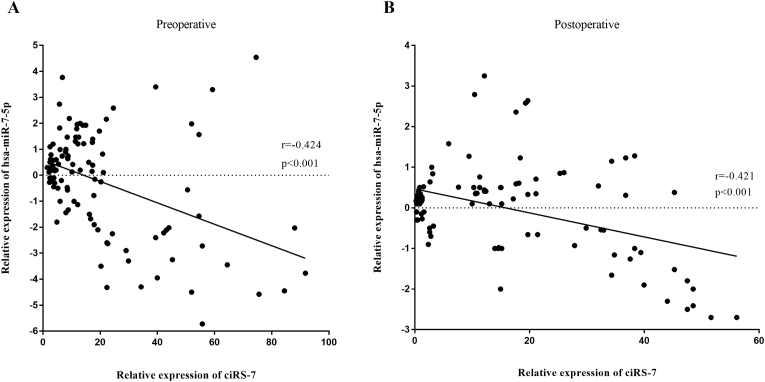
Relative expression of ciRS-7 and its potential target, hsa-miR-7-5p, in plasma samples from patients with epithelial ovarian cancer (EOC) FIGO stage I-IV. **(A)** Correlation between circulating ciRS-7 expression and hsa-miR-7-5p levels in preoperative plasma EOC samples (p < 0.001; Spearman rank correlation test: r = −0.423; n = 111). **(B)** Correlation between circulating ciRS-7 expression and hsa-miR-7-5p levels in postoperative plasma EOC samples (p < 0.001; Spearman rank correlation test: r = −0.421; n = 111).

The expression level of ciRS-7 correlated negatively with hsa-miR-7-5p in plasma. In EOC FIGO stage I-IV patients (n = 111), ciRS-7 showed a negative correlation with hsa-miR-7-5p in pre-(r = −0.423, p < 0.001) and postoperative (r = −0.421, p < 0.001) periods (Fig. 5A and B). Consequently, the expression of ciRS-7 and its putative target hsa-miR-7-5p had a strong inverse relation in human EOC with FIGO stage I-IV, thereby suggesting that the essential roles of ciRS-7 acts as a sponge of hsa-miR-7-5p and thus inhibits its oncosupressive activity most likely for advanced stage of EOC.

## 4 Discussion

Despite progress in the treatment of OC, this malignant neoplasm remains the leading cause of death among tumors. Over the past decade, there has been an upward trend in morbidity rates in almost all countries, along with modest reductions in mortality and 5-year survival [1,2]. In 70–80% of patients, OC is diagnosed at an advanced stage [14]. Early diagnosis of OC is difficult due to the asymptomatic nature of OC in the early stage and the lack of sensitive screening methods. CA 125 is the main biomarker used for the diagnosis of OC [15]. However, in the early stages of ovarian cancer, the content of CA 125 in the blood does not undergo significant changes. At the same time, an increased concentration of this biomarker can indicate diseases such as autoimmune diseases, infections, other tumors (e.g. breast cancer, lung cancer, and pancreatic cancer), etc. [16,17]. The above list of diseases indicates that it is impossible to make an accurate diagnosis only on the basis of this biomarker. Circulating cirсRNAs have great potential for the diagnosis and prognosis of human tumors. The covalently closed loop structure without 5′–3′ polarity or polyadenylated tail confers great stability upon circRNAs in human biofluids, preventing them from being degraded by RNases [18]. As a result, the average half-life of circular circRNAs in biofluids is much longer than that of mRNAs or miRNAs [19]. In addition, circulating circRNAs have significant diagnostic and prognostic value, not only because of the ease of detection but also because of the manifestation of specific tissue expression/stage of development of the disease, including tumors [5].

In this study, pre-and postoperative expression levels of plasma ciRS-7 in 47 EOC patients with FIGO stage IA-IIB and 64 EOC patients with FIGO stage IIB-IV were first screened using qRT-PCR arrays. We found that circulating cirRS-7 was significantly increased in EOC patients with FIGO I-IV in the preoperative period than in the healthy controls. Furthermore, we saw that in the postoperative period the expression levels of plasma ciRS-7 were significantly lower in the EOC FIGO stage IA-IIA patients group while and in the EOC FIGO stage IIB-IV patients group there was only a slight decrease in the expression levels of circulating ciRS-7 than in the healthy controls. In addition, using ROC curve analysis, we demonstrated that the circulating ciRS-7 had high accuracy in EOC diagnosis, especially in patients with FIGO IIB-IV stage disease in pre-and postoperative periods where AUC was 0.92 and 0.88, respectively. To the best of our knowledge, this is the first EOC study to specifically focus on circulating ciRS-7.

Previous studies have identified that ciRS-7 is an important non-coding RNA in regulating the malignant behaviors as invasion and metastasis in multiple cancers, including esophageal squamous cell carcinoma (ESCC), triple-negative breast cancer (TNBC), NSCLC, and OC [8,9,20,21]. For instance, San et al. demonstrated that ciRS-7 could effectively accelerate tumor growth and metastasis of ESCC through targeting the miR-876–5p/MAGE-A axis. The authors also showed that high expression levels ciRS-7 are associated with poor clinicopathological characteristics in ESCC patients [20]. In another study, ciRS-7 promoted OC cell growth, colony formation, migration, and invasion by sponging miR-641 to up-regulate ZEB1 and MDM2 expression. Wherein ciRS-7 expression was significantly associated with the TNM stages, lymph node metastasis status, and OS rate in OC patients [9]. In this study, we find that expression was positively correlated with lymph node metastasis, distant metastasis, and was an independent factor predicting survival in EOC patients. Our data demonstrate that high expression of circulating ciRS-7 was significantly associated with the lymph node metastasis status. Moreover, high expression of circulating ciRS-7 in plasma has a positive correlation with liver and lung metastasis from EOC. Therefore, we further visualized the ROC curve and found that the AUC was 0.89, 0.84, and 0.76, which indicated that circulating ciRS-7 had a very high diagnostic value and demonstrated that this circRNA displayed considerable accuracy in discriminating patients with metastasis from the patients without metastasis. Future work will be required to study the role of ciRS-7 in metastasis from EOC *in vitro* and *in vivo*. In addition, to analyze the association of circulating ciRS-7 expression with prognosis, the Kaplan-Meier method and log-rank test showed that higher ciRS-7 expression showed a shorter OS compared to lower ciRS-7 expression in EOC patients. Wherein patients with EOC advanced stage in the higher-expression group of ciRS-7 had a poorer OS than in the patients with EOC early stage. Multivariate Cox analysis showed that higher circulating ciRS-7 was an independent poor prognostic factor for patients with EOC advanced stage. Thus, the significantly higher ciRS-7 expression in OC cells and tissues with poor clinicopathological characteristics in research of Zhang et al. may explain why in our study was found that significantly higher circulating ciRS-7 expression in plasma correlates with FIGO stages, lymph node and distant metastasis status, and poor prognosis in EOC patients [9].

MicroRNAs (miRNAs) are 18–22 nucleotide endogenous non-coding RNAs that regulate gene expression at the post-transcriptional level by interacting with 3′-untranslated regions (3′-UTR) of mRNA-targets [22]. It has been proven that miRNAs are involved in the molecular mechanism of the development and progression of various human tumors, including OC [[23], [24], [25], [26], [27]]. Given their importance in the regulation of gene expression, there is great interest in understanding the regulatory mechanisms that can regulate miRNA function. To date, circRNAs have been shown to play a crucial role in the regulation of gene expression, in part by inhibiting miRNA activity [28]. It is known, that ciRS-7 acts as an oncogene and promotes tumor progression through competitively inhibiting oncosuppressive miR-7 in various types of human tumors [29]. For instance, Li et al. showed that overexpression of ciRS-7 inhibited the tumor-suppressive effects of miR-7 and contributed to malignant progression in ESCC via regulation HOXB13-mediated NF-κB/p65 pathway [30]. Other results demonstrated that overexpression of ciRS-7 could inhibit miR-7-mediated tumor suppression via antagonizing miR-7-mediated phosphatase and tensin homolog deleted on chromosome 10 (PTEN)/phosphoinositide-3-kinase (PI3K)/AKT pathway in gastric cancer [31]. Zhang et al. found that the high expression of ciRS-7 promoted the proliferation and inhibited apoptosis of NSCLC cells to up-regulated EGFR, CCNE1, and PIK3CD via sponging miR-7 [32]. However, the function of the ciRS-7-miR-7 network in OC is not understood. Given the roles of ciRS-7 as miR-7 sponge and used the online tools targetScan, miRanda, and circular RNA Interactome to predict the possible miRNA binding to ciRS-7 and hsa-miR-7-5p was found to be the best potential candidate. As result, the current study showed that ciRS-7 correlated negatively with hsa-miR-7-5p in pre-and postoperative plasma of EOC patients. These results further suggest that ciRS-7 may play a significant role in the development and progression of EOC by acting as a hsa-miR-7-5p sponge. Future studies *in vitro* and *in vivo* may address whether dual targeting ciRS-7 and hsa-miR-7-5p may provide a novel therapeutic strategy to suppress the progression of EOC.

In summary, ours is the first study to systematically interrogate the clinical significance of circulating ciRS-7 in EOC, and we provide comprehensive evidence that it may act as an oncogenic circRNA, as well as a non-invasive diagnostic and prognostic biomarker in EOC, particularly EOC with advanced stage.

## 5 Conclusions

We verified that increased the expression levels of circuiting ciRS-7 is a prospective candidate non-invasive biomarker for EOC diagnosis, prognosis and correlate positively with disease activity and behavior in patients with advanced stage of EOC. Moreover, ciRS-7 may participate in the molecular mechanism of EOC by sponging hsa-miR-7-5p. Confirming the potential role of ciRS-7 in the oncogenesis of EOC and confirming it as a non-invasive biomarker in early diagnosis and prognosis of EOC requires more clinical trials and *in vitro* and *in vivo* studies.

## Funding

None.

## Consent to participate

Written informed consent was obtained from all individual participants included in the study.

## Ethical approval

All plasma samples were collected with approval from all the patients and the ethics committee of Republican Clinical Oncological Dispensary.

## CRediT authorship contribution statement

**Aferin Beilerli:** Conceptualization, Funding acquisition, Formal analysis, Data curation, conceptualized and designed the study. All authors participated in the acquisition, analysis and interpretation of the data. **Sema Begliarzade:** Conceptualization, Funding acquisition, Formal analysis, Data curation, conceptualized and designed the study. All authors participated in the acquisition, analysis and interpretation of the data. **Albert Sufianov:** Writing – original draft, drafted the manuscript. **Tatiana Ilyasova:** Writing – original draft, drafted the manuscript. **Yanchao Liang:** Writing – original draft, drafted the manuscript. **Ozal Beylerli:** contributed to critical revisions of the manuscript. All authors agreed on the journal to which the article would be submitted, gave final approval for the version to be published, and agreed to be accountable for all aspects of the work.

## Declaration of competing interest

The authors declare they have no conflict of interest.
